# Studying the Factors Affecting Tool Vibration and Surface Quality during Turning through 3D Cutting Simulation and Machine Learning Model

**DOI:** 10.3390/mi14051025

**Published:** 2023-05-10

**Authors:** Quang Ngoc The Ho, Thanh Trung Do, Pham Son Minh

**Affiliations:** 1 Faculty of Mechanical Engineering, HCMC University of Technology and Education, Ho Chi Minh City 71307, Vietnam; quanghnt.ncs@hcmute.edu.vn (Q.N.T.H.); minhps@hcmute.edu.vn (P.S.M.); 2 Faculty of Engineering and Technology, Nguyen Tat Thanh University, Ho Chi Minh City 70000, Vietnam

**Keywords:** cutting process, surface roughness, cutting force, machine learning

## Abstract

Nowadays, machining products, especially by turning methods, are more and more popular and require high-quality. With the development of science and technology, especially numerical computing technology and control technology, the application of these technological achievements to improve productivity and product quality has become increasingly essential. This study applies a simulation method considering the affecting factors of the vibration of the tool and the surface quality of the workpiece during turning. The study simulated and analyzed the characteristics of the cutting force and oscillation of the toolholder when stabilizing; at the same time, the study also simulated the behavior of the toolholder under the effect of cutting force and determined the finished surface quality through simulation. Additionally, the study utilized a machine learning model to examine the relationship between the toolholder length, cutting speed, feed rate, wavelength and surface roughness. The study found that tool hardness is the most crucial factor, and if the toolholder length exceeds the critical length, it leads to a rapid increase in roughness. In this study, the critical toolholder length was determined to be 60 mm, and this resulted in a corresponding surface roughness (Rz) of approximately 20 µm.

## 1. Introduction

Turning machining method is still a traditional method widely used in workpiece machining. This is a method performed on a lathe in which the workpiece rotates and the tool reciprocally moves to produce the cutting process. During the cutting process, there are collisions and interactions between the workpiece and the tool, creating cutting forces. This cutting force acts on the workpiece, the tool and the holder. However, due to the workpiece and the toolholder’s elasticity, there are changes in their relative positions causing vibrations. This vibration causes machining errors and reduces the surface quality of the part after machining. That is the reason why it has become a noticeable topic in the manufacturing industry and academia for many years. Since the late 1950s, many research studies have been conducted to solve the chatter problem. They have studied how to detect, identify, avoid, prevent, reduce, control, or suppress chatter. Vibration has many causes and is influenced by many factors, including the mechanical system, the rigidity and precision of the machine, the tool, the workpiece and the fixture. The cutting mode also affects the stability and vibrational characteristics of the system. There are many published papers analyzing mechanical phenomena and mathematical models of this phenomenon [1,2].

R. Mahdavinejad used the finite element method and commercial software ANSYS to analyze the stability of the machining system. This study has described the vibration phenomenon of the machine and the tool, as well as built the stability diagram [3]. C. S. Kumar et al. studied the turning process of AISI 1045 steel with multi-layer TiAlCrN-coated cutting tools. The study used the software Abaqus/Explicite with the destruction model based on energy destruction. The results showed that applying a coating to the cutting tool not only reduced friction and temperatures at different cutting speeds but also significantly impacted the mechanism of chip formation. Both coated and uncoated cutting tools generated a secondary shear plane (SSP) during machining. However, the SSP only appeared at higher cutting speeds when using the coated cutting tool. The finite element model that was proposed can accurately predict the formation of secondary serrated teeth and instability [4]. M. S. Hajmohammadi and M. R. Movahhedy examined the impact of temperature on vibration while turning. By analyzing workpiece parameters, they evaluated the influence of temperature on friction, plastic deformation, heat generation, and damping. This study indicated that changes in temperature will affect the steady-state graph [5]. C. Brecher et al. developed a 3D simulation model for turning, which incorporated cutting force and cutting process simulations based on the workpiece and tool geometry parameters. The study’s findings indicated that cutting with defined and undefined cutting edges, as well as sheet and bulk metal forming, were critical processes in the manufacturing industry. These processes allowed for the production of a diverse range of metal parts [6].

Moreover, after performing simulations to determine vibration characteristics during turning, a number of research works have conducted experiments to compare and adjust parameters between the simulated model and reality. A. K. Parida and K. Maity used Deform software to simulate the hot turning of Inconel 625 material with different cutting parameters. It showed that the hot turning temperature had an effect on cutting force, tool wear and tool life. In addition, simulation and experimental results were similar to chip formation and shear force [7]. Meanwhile, O. B. Abouelatta and J. Madl predicted the surface roughness based on cutting parameters and tool vibrations in turning operations. The study conducted experiments to collect data and used experimental planning tools SPSS and MATLAB to determine the influence relationship of the factors of tool feed quantity, and depth of cut to the quality of the surface of the parts after machining [8].

Several authors have applied machine learning to study process parameters. C. Zhu et al. used a neural network algorithm to predict the drilling force for high volume fraction SiCp/Al composite. The result showed that the predicted drilling force at different drill rotation speeds and feed rates, based on the improved neural network algorithm, is consistent with the experimental drilling force, and the prediction model has high accuracy [9]. J. G. Parmar et al. predicted the milling process parameters using an artificial neural network (ANN) with 5 input neurons, 6 hidden layers and 6 output neurons. In their research, AISI1020 steel was chosen as the work material, and a solid carbide end milling cutter was used as the cutting tool. They concluded that the ANN model can efficiently predict the multi-response parameters of the end milling process for any selected materials without experimentation cost and time [10]. X. Zhou et al. investigated the classification of tool wear during high-precision turning of copper, based on time series images processed by the convolutional neural network. The authors used the Gramina Angular Summation Fields method to convert one-dimensional cutting force signals into two-dimensional time series images, which were then classified by a modified AlexNet network according to the type of tool wear. The authors found that the overall accuracy of the classification for the four categories of tool wear was approximately 90%, based on the prediction results [11].

In general, many studies have applied numerical and experimental methods to characterize the vibration phenomenon and surface quality in the turning process related to many factors such as cutting force, workpiece deformation, and operating technology parameters [12,13,14,15,16]. Typically, investigations into the surface quality of post-machined parts rely on experimental techniques [1,2,8]. While this method provides an intuitive and precise model, it comes with significant drawbacks. The implementation of such methods can be expensive and require high-quality test and measurement equipment. Additionally, confounding factors can impact experimental results and models, limiting their application to specific material and machine cases. Consequently, the development of a new and more costly model is necessary when exploring alternative scenarios. Nowadays, with the development of computer technology and numerical methods, simulations have become an essential tool in research [17,18,19,20]. Therefore, in this study, the authors have utilized a simulation method to simulate the turning process while focusing on using numerical calculations to analyze various factors that affect the oscillation of the tool. The characteristic parameters of oscillation, such as amplitude and oscillation period, have been derived. Additionally, the study investigates the factors that impact the surface quality of finished parts, specifically the surface roughness. To achieve this, the neural network model has been implemented to establish the relationship between the feed rate f (mm), cutting speed v (m/min), stiffness of the toolholder through its length l (mm), workpiece surface undulation wave as wavelength s (mm), and the finished surface quality as surface roughness Rz (µm). In this paper, Rz is used instead of Ra to calculate the surface roughness because the simulation results provide the undulating profile after turning, making it easy and accurate to determine the 5 highest peaks and 5 deepest valleys. Additionally, Rz and Ra are theoretically related, with Rz being approximately 4 times that of Ra.

## 2. Simulation Method

### 2.1. The Simulation Model

**Figure 1:**
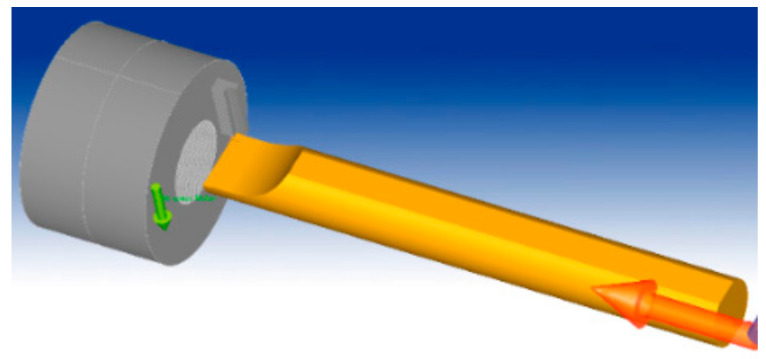
The model of the workpiece and tool.

**Figure 2:**
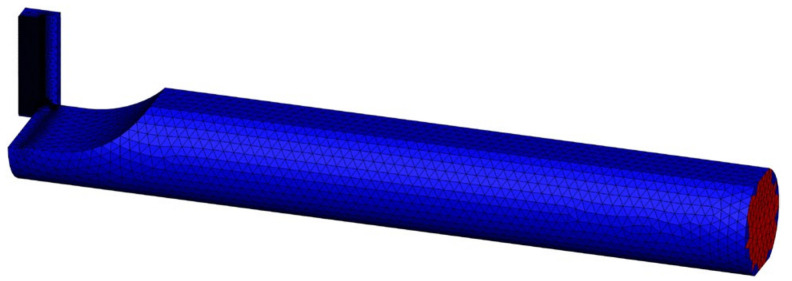
The mesh and boundary conditions.

In the cutting process, vibration is a common occurrence and can be caused by many factors. However, the primary cause of vibration is the rigidity of the technology system, mainly the elastic deformation of the tool and the workpiece [8,16,20]. To investigate the factors that affect the vibration and stability of the system, this study employs a numerical calculation tool. Specifically, a 3D model is used as shown in Figure 1 and Figure 2 for simulating the cutting process in both stable and oscillating tool conditions. The force value over time is then analyzed to assess the stability of the tool based on the dispersion of the force value around the nominal force value. Additionally, the study uses a simulation method to extract detailed surface quality data after machining. The workpiece, tool and cutting parameters are shown in Table 1 [1,8]. The selected parameters were based on the cutting conditions with the workpiece material being Al_6061 aluminum and the tool material being carbide. The research model was machining a hole with a diameter of Ø30 on a lathe machine with cutting speeds ranging from 40 to 125 m/min. The parameters of the tool feed rate and cutting depth were selected according to the manufacturer’s recommendations. In this study, the deformed tool model with the Johnson-Cook destructive and plastic deformation model is used for simulation with the hole-cutting process [2,21].

**Figure 3:**
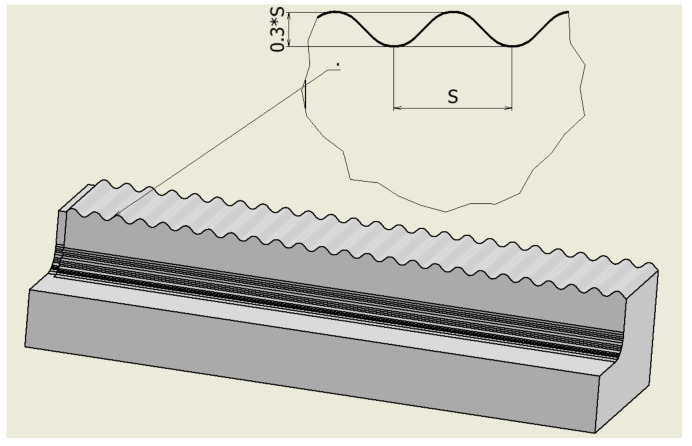
The undulating workpiece surface.

**Figure 4:**
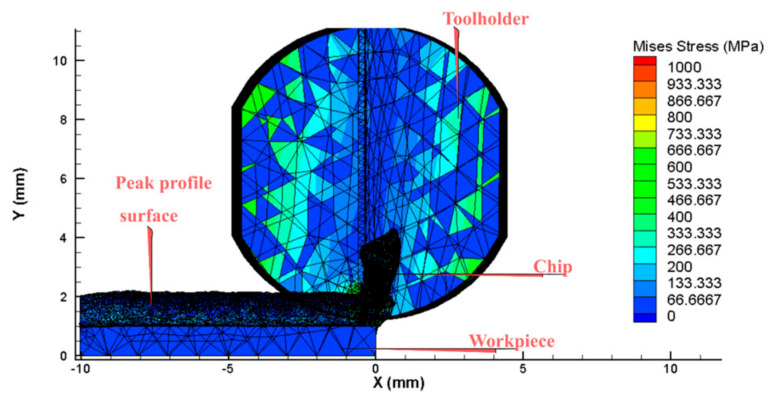
The peak of the finished surface profile.

**Figure 5:**
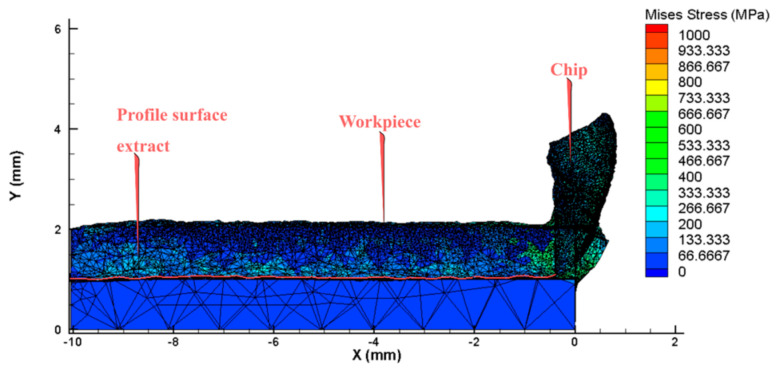
The curve of the finished surface profile.

Previous studies mainly simulated the case of a flat workpiece and an absolute hard toolholder [1,4,22]. However, in this study, the workpiece has an undulating surface, as shown in Figure 3, and the toolholder is subject to elastic deformation. After simulating the cutting process using a 3D model, the study focuses on the extraction of cutting force in the Y direction. This direction is chosen because it leaves surface undulations on the workpiece when the toolholder is deformed. Then, the 5 highest peaks and 5 lowest peaks of the cutting force values are identified as shown in Figure 4 and Figure 5. These 10 values of cutting force are used to determine the deformation and displacement of the tool relative to the workpiece. Specifically, the study applies these values to the toolholder to determine the average displacement, which represents the surface roughness value of the finished surface product after turning [8,15].

### 2.2. The Machine Learning Model

**Figure 6:**
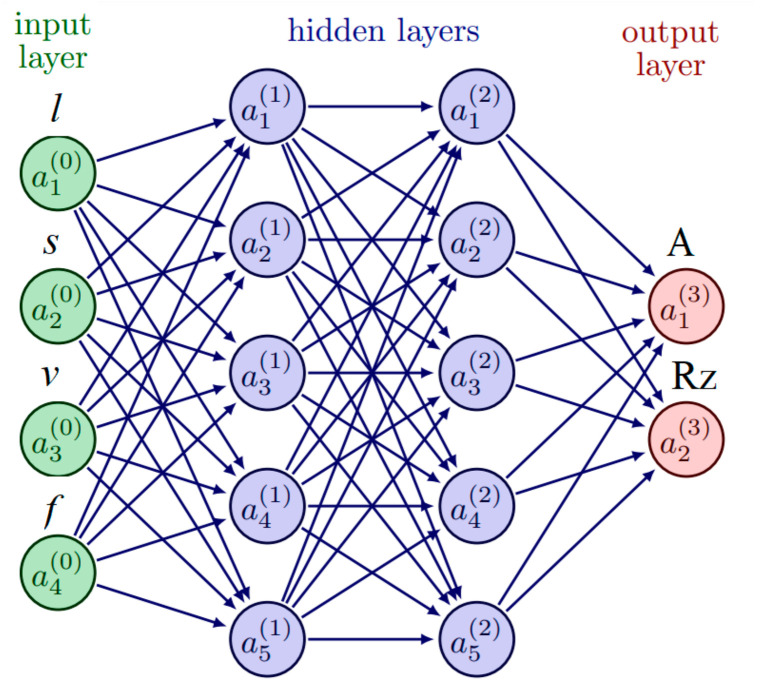
Neural network with four inputs and 2 outputs.

This study employs a neural network, which is comprised of interconnected nodes that form a computational model based on input data factors and predict the output values. The model employed in this study has four input parameters, namely, toolholder length (l), wavelength (s), cutting speed (v), and feed rate (f). Additionally, it has two output values such as surface roughness (Rz) and cutting force (A), as shown in Figure 6. Additionally, in this case, the model used two hidden layers, each with five neurons and the ReLU (Rectified Linear Unit) activation function, to learn complex representations of the input data. The connections between neurons in adjacent layers have corresponding weights, which are adjusted during the training process to minimize the error between the model’s predictions and the target values. Backpropagation is used to update the weights and biases based on the derivatives of the loss function. Once the training process is complete, the model can make more accurate predictions for new data based on the learned parameters [19,21,23].

## 3. Results and Discussion

### 3.1. The Effect of Toolholder Length

When the toolholder is completely rigid, the cutting force acting on the tool and the workpiece remains relatively constant, regardless of whether the workpiece surface is flat or undulating. This is because, in a rigid toolholder, the distance between the tool and the workpiece remains constant and any changes in cutting force are due to variations in material thickness caused by the undulations. However, achieving an absolutely rigid, non-deforming toolholder is an ideal condition that is difficult to achieve in practice, as the toolholder is typically subject to elastic deformation.

**Figure 7:**
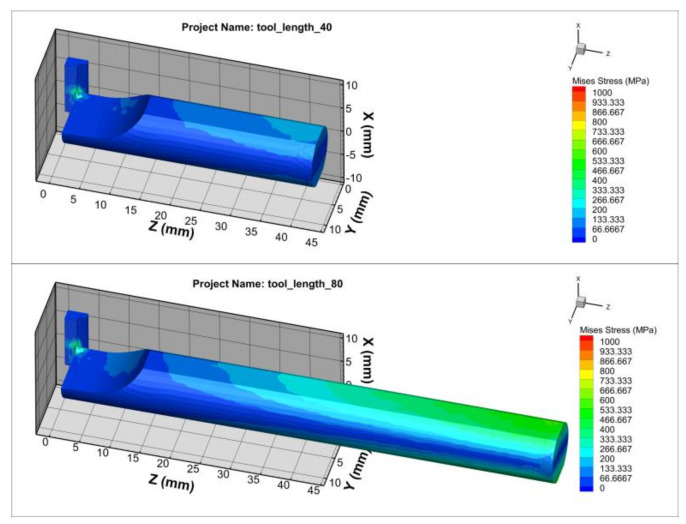
The Von Mises stresses for the toolholder lengths of 40 mm and 80 mm.

**Figure 8:**
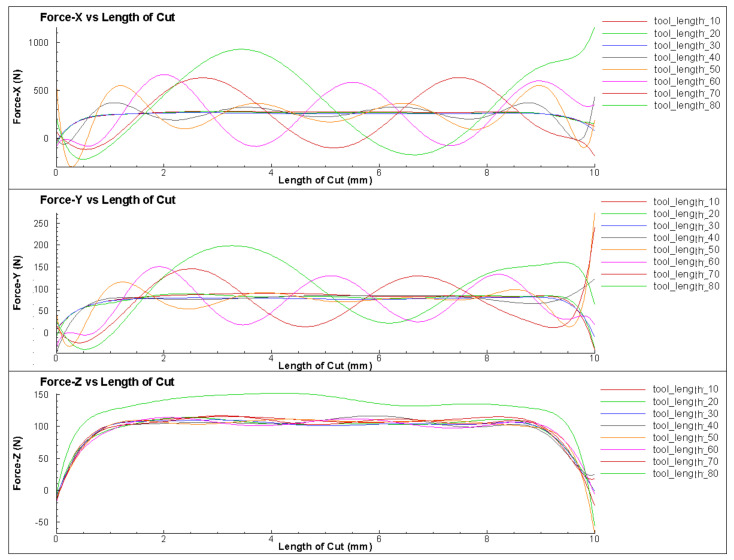
The cutting forces for different toolholder lengths from 10 to 80 mm.

The stiffness of the deformation of the toolholder varied depending on its length. When turning with an elastically deformed toolholder, the tooltip shifted position relative to the workpiece, resulting in a reduction in cutting depth. As the cutting depth decreased, the cutting force also decreased, which reduced the deformation of the toolholder. Figure 7 shows the effect of the toolholder length on the Von Misses stress relating to the variation in cutting force [1,6]. During machining processes, the elastic deformation of the tool caused the tip to shift in relation to the workpiece, thereby leading to a decrease in cutting depth. Consequently, the cutting force was also reduced as the cutting depth decreased. With the decrease in cutting force, the toolholder underwent less deformation and returned to its original state. In the turning case, the amplitude and frequency of cutting force varied at different toolholder lengths, as shown in Figure 8. It shows that longer toolholders resulted in decreased stiffness deformation, leading to greater deformation of the tool and gradual increases in amplitude, period and force. However, the average cutting force value remained constant, in accordance with theoretical and experimental calculations. For toolholder lengths below 50 mm, the cutting force fluctuated only slightly, remaining almost constant. However, when the toolholder length exceeded 50 mm, the cutting force fluctuation became significantly larger, with clear periods and amplitudes (Table 2). When the toolholder length was 60 mm, the cutting force fluctuated between 20 N and 120 N, corresponding to a surface cycle of approximately 2 mm.

**Figure 9:**
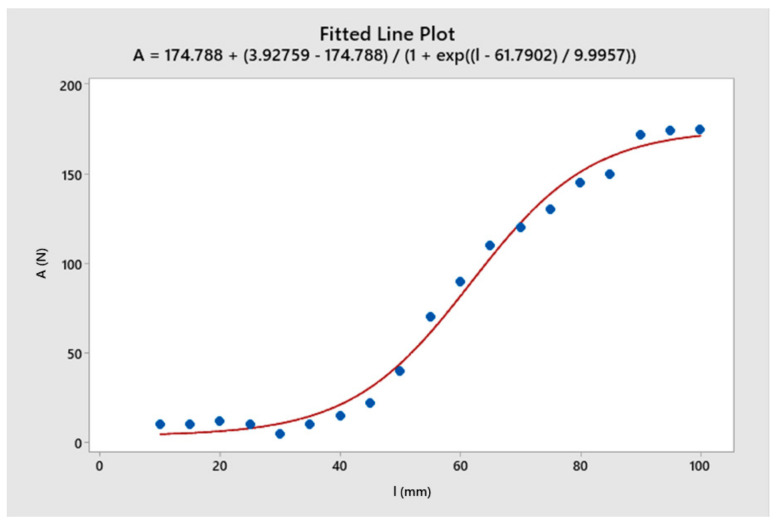
The regression analysis of cutting force as a function of the toolholder length.

**Figure 10:**
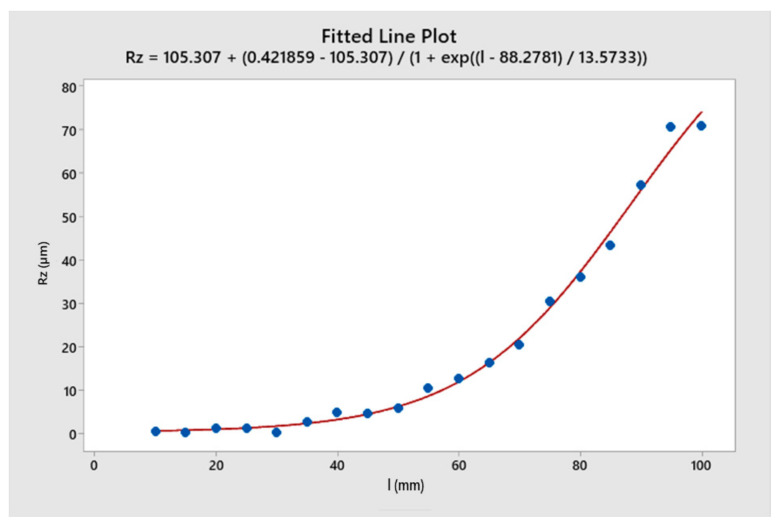
The regression analysis of surface roughness as a function of toolholder length.

The regression analysis of the cutting force and the surface roughness that depended on the length of the toolholder is shown in Figure 9 and Figure 10, respectively. For the toolholder with a length of less than 50 mm, the cutting force and the surface roughness were minimal. This could be explained by the fact that when the toolholder length was short, the toolholder deformation was small enough to maintain a negligible change in the relative position between the tool tip and the workpiece. As a result, the cutting force reached a steady state. This finding had significant implications for technologists who needed to identify the optimal toolholder length to achieve a steady state of the cutting tool [2]. In the length range of 50 mm to 85 mm, both the cutting force and the surface roughness increased rapidly. This could be attributed to the decreasing elastic stiffness of the toolholder. At this point, the tool deformed and the relative position of the tool and workpiece changed, leading to a decrease in cutting force. The elastic force of the toolholder then caused the tool to tend to return to its original position. In this case, the cutting force and the elastic force of the toolholder were equivalent, resulting in large oscillations. On the other hand, when the toolholder length exceeded 85 mm, the oscillation in cutting force and surface undulation tended to change less. This could be explained by the fact that when the toolholder was long enough, the tool deformed significantly, but the elastic force of the toolholder was small enough to establish a new position.

### 3.2. The Effect of Wavelength

**Figure 11:**
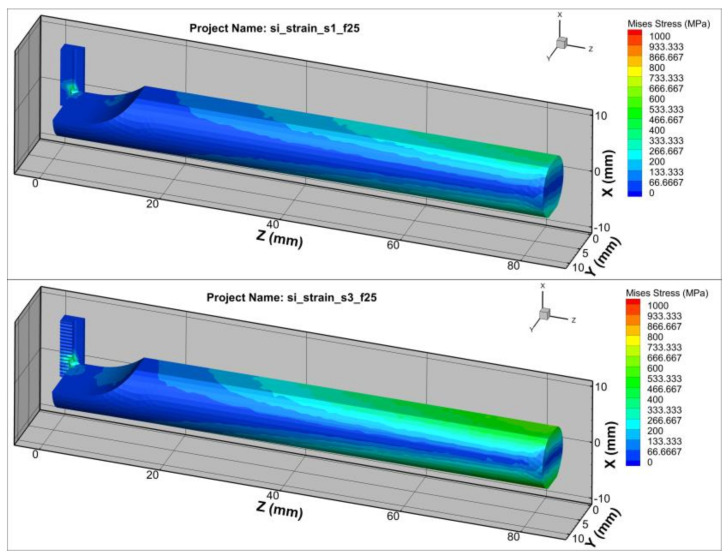
The Von Mises stresses for the wavelengths of 0.01 mm and 0.2 mm.

**Figure 12:**
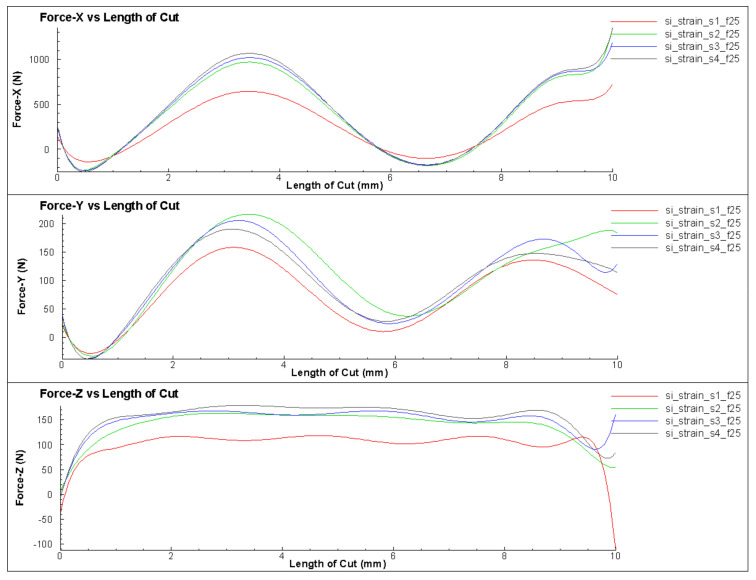
The cutting forces for different wavelengths of 0.01, 0.05, 0.15 and 0.2 mm.

According to the cutting theory, the vibration phenomenon during turning is caused by the influence of the undulating workpiece surface and the tool’s oscillation [2,15]. Therefore, this section investigated the effect of the wavelength on the change of cutting force during turning, as shown in Figure 11. In order to determine the characteristics of changes in cutting force and the factors that influence these changes, four cases were selected for simulation as shown in Figure 12. The force amplitude and oscillation period were the characteristic parameters for the change in cutting force. The oscillation period change in the cutting force value was calculated not only with time but also with the cutting length as the workpiece’s moving length to compare different cases easily. The model considered 21 different wavelengths (from 0 to 0.2 mm) to observe the change in force value, as shown in Table 3.

**Figure 13:**
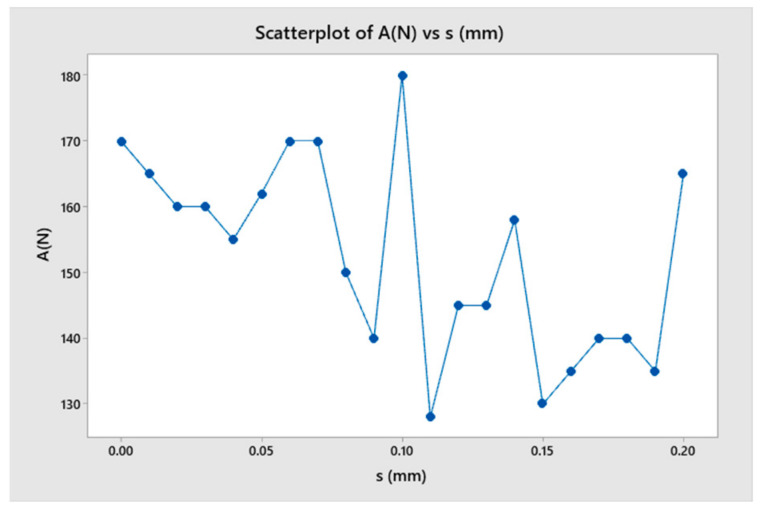
The cutting force as a function of wavelength.

**Figure 14:**
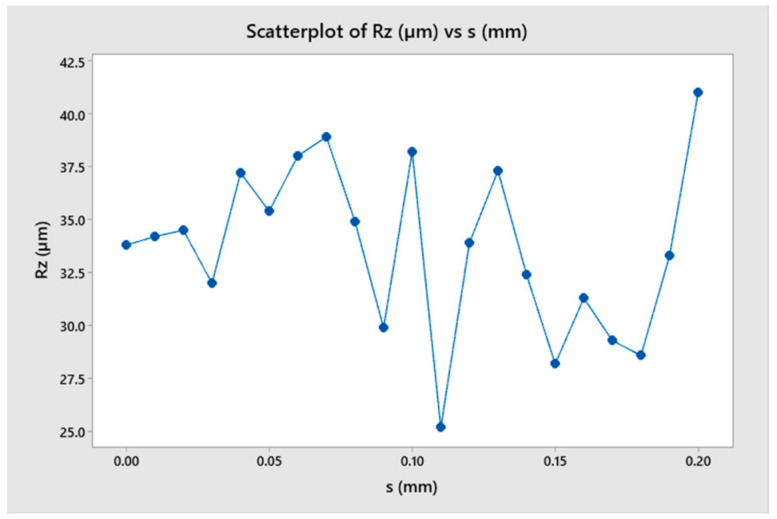
The surface roughness as a function of wavelength.

The results showed that although the surface undulation corresponded to different roughness levels, it did not greatly influence the amplitude and frequency of oscillation of the cutting force (Figure 13 and Figure 14). Figure 13 shows the case of a flat workpiece, while the remaining cases corresponded to surface undulating waves with different values. When the wavelength was large, the force amplitude changed, which was explained by the high undulating wave amplitude leading to a larger cutting volume, resulting in a larger amplitude of change in force. However, the force change period remained almost constant, which could be explained by the force change period being mainly determined by the length and the stiffness of the toolholder.

### 3.3. Effect of the Cutting Speed

**Figure 15:**
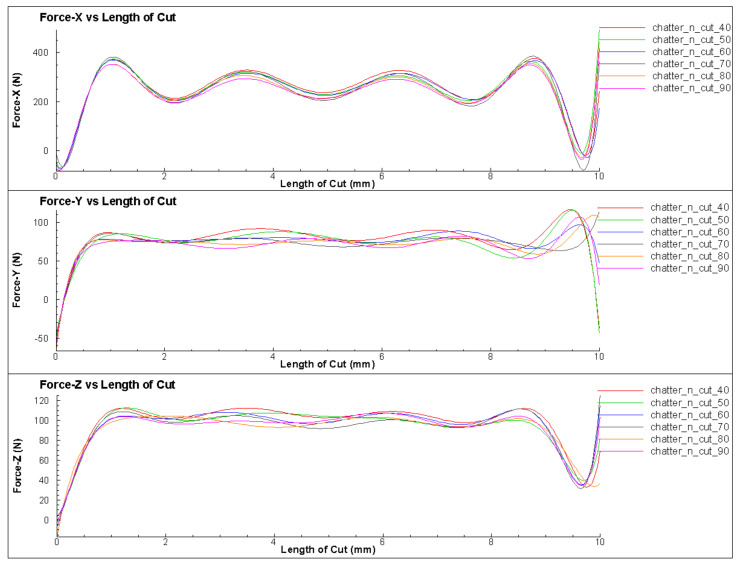
The cutting forces for different cutting speeds with X axis is a cutting length.

**Figure 16:**
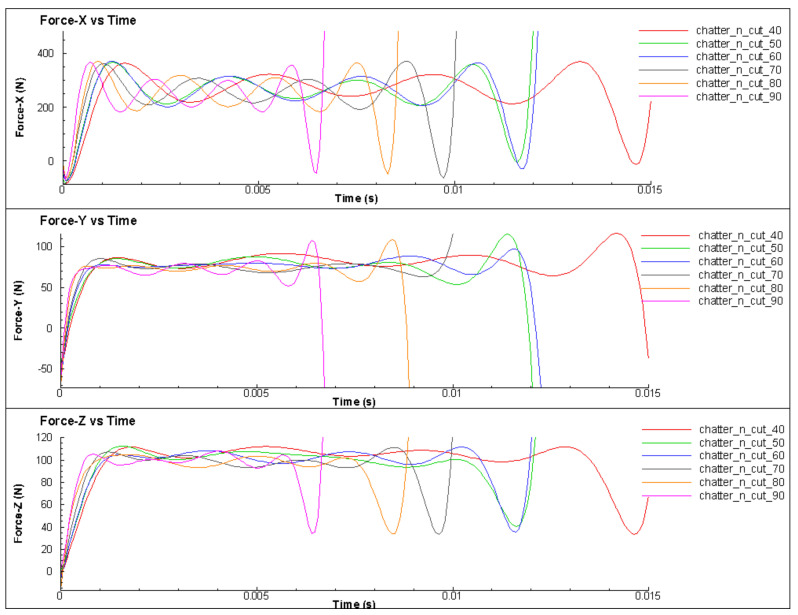
The cutting forces for different cutting speeds with X axis is a cutting time.

**Figure 17:**
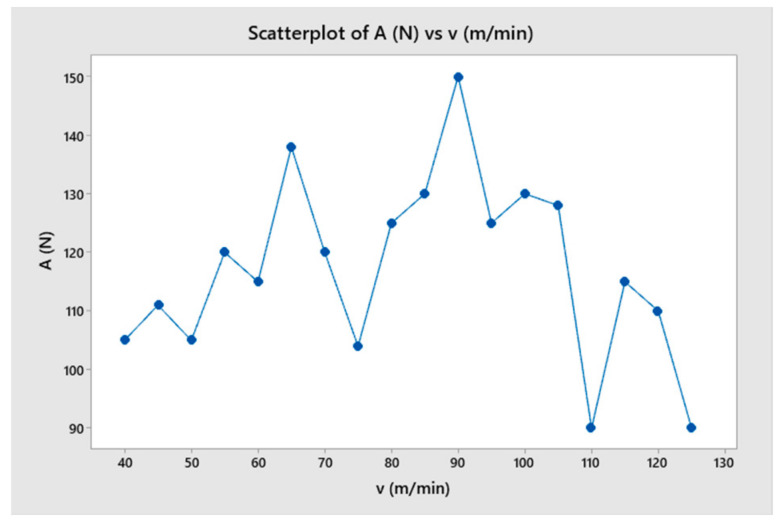
The cutting force as a function of cutting speed.

**Figure 18:**
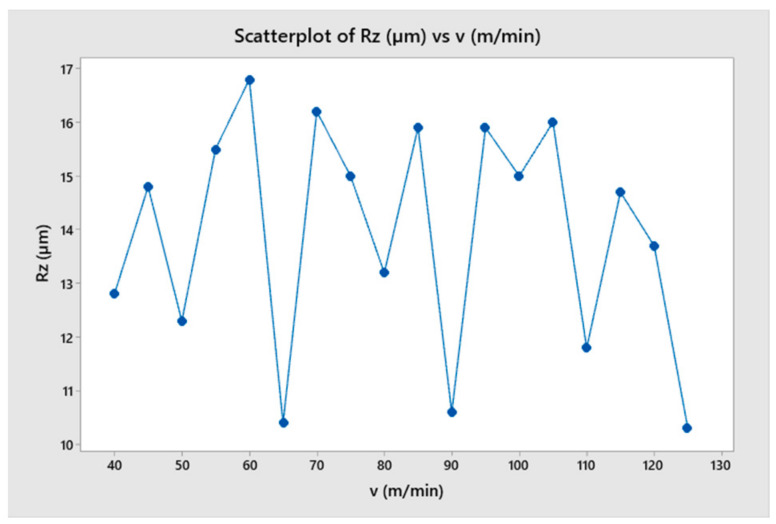
The surface roughness as a function of cutting speed.

The effect of cutting speed on the change of cutting force in the turning process was investigated in this section. This study involved turning under the same cutting conditions, with different cutting speeds ranging from 40 m/min to 90 m/min. The results show that at different cutting speeds, the amplitude and period of change of the cutting force remained almost constant, as shown in Figure 15 and Figure 16. This could be attributed to the fact that, when all other parameters were held constant, the cutting speed only changed the simulation time, and not the cutting condition. To compare the difference in the force change cycle, the time-varying cutting force cycle was considered. However, the simulation study shows that the variation of the cutting force and its relationship with the surface quality of the machined part depended very little on the cutting speed. The variable values and trends were not clear, as shown in Figure 17 and Figure 18. When comparing the cutting force graphs at different cutting speeds (v = 40, 50, 60, 70, 80 and 90 m/min), there was a difference in terms of time as shown in Table 4. However, when considering the cutting length, the graphs were equivalent. It is possible that the effect of the inertia force was not significant due to the relatively small size of the simulated workpiece.

### 3.4. Effect of the Feed Rate

**Figure 19:**
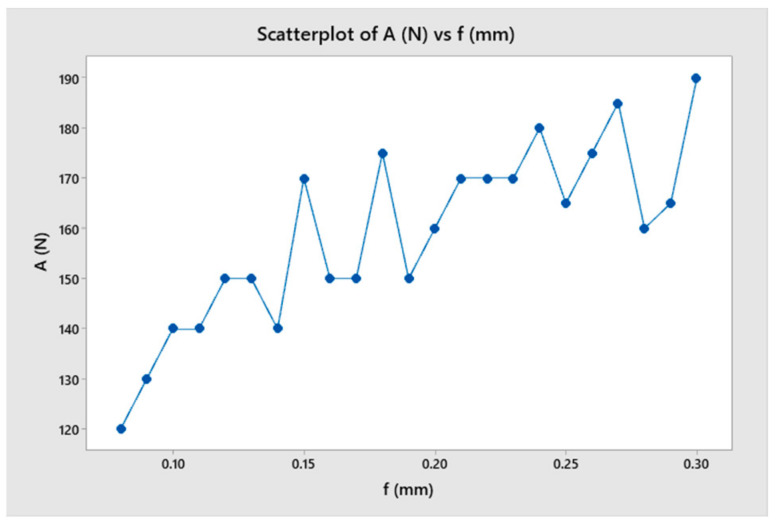
The cutting force as a function of feed rate.

**Figure 20:**
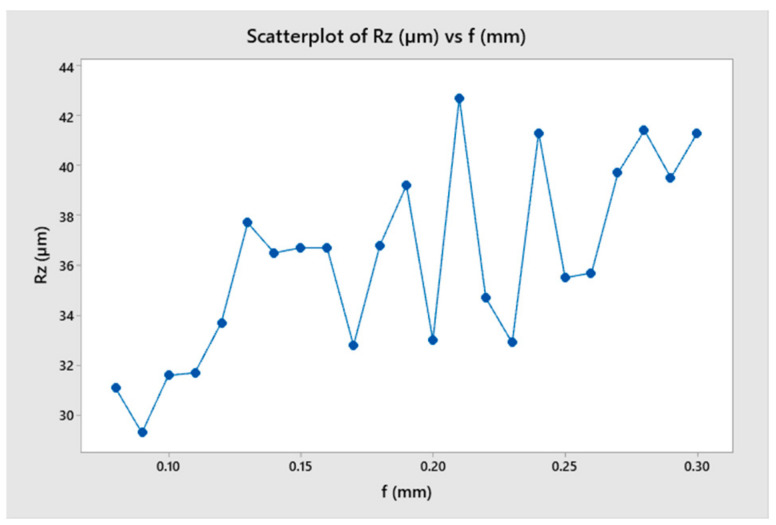
The surface roughness as a function of feed rate.

A study was conducted to determine the stable range of cutting feed rates for a toolholder with a constant length. The study also analyzed the tool’s oscillation amplitude and period and how it affected the surface quality of the machined part. Table 5 summarizes the results and indicates that cutting force increased as the feed rate increased from 0.09 to 0.18 mm, but the change in cutting force over time was relatively small compared to the range of 0.21 to 0.30 mm. The result shows that the amplitude of cutting force variation was highest for feed rates of 0.21 and 0.30 mm. This could be explained when the frequency of oscillation of the toolholder was close to the frequency of undulation of the workpiece (Figure 19 and Figure 20).

### 3.5. Effect of Multi Factors

**Figure 21:**
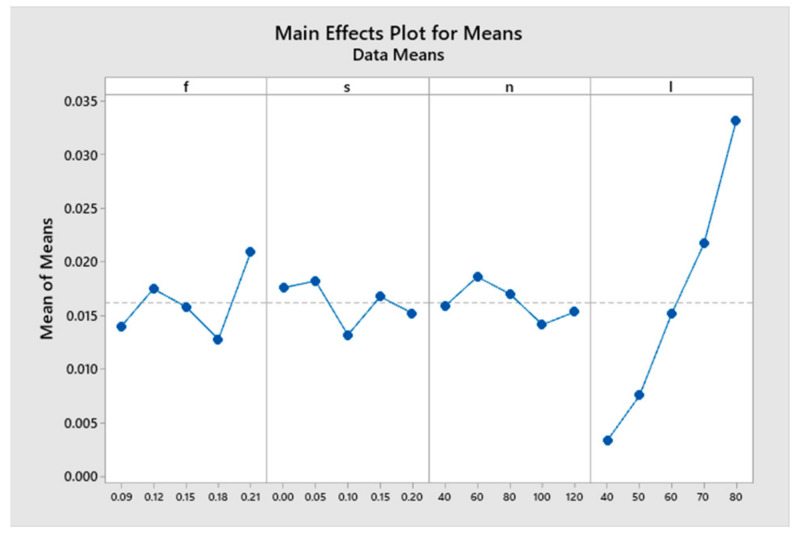
The S/N ratio in Taguchi method.

The results were obtained on the effects of feed rate, cutting speed, wavelength and toolholder length on the changing value of cutting force and surface roughness after turning as shown in Table 6. Additionally, each parameter had five levels and the orthogonal matrix was 25 (Taguchi OA L25). After the factors affecting the oscillation characteristics of the toolholder and the factors affecting the surface quality of the finished part were studied by simulation method, it found the oscillation characteristics of the toolholder with amplitude and period of oscillation by time and by cutting length as shown in Figure 21. With the changing characteristics of this cutting force, it became the basis for studying the application of external forces acting on the toolholder to reduce the oscillation of the toolholder and improve the surface quality of the part after machining as shown in Table 7. The results show that all of the considered parameters in this study affect the quality of the machined surface, with the toolholder length having the greatest influence, followed by the feed rate, wavelength and cutting speed, respectively. This finding is consistent with previous studies on the parameters that affect the finished surface roughness [8,13].

Moreover, applying a machine learning model to determine the relationship between technological parameters of toolholder length (l), wavelength (s), cutting speed (v), feed rate (f) and surface roughness (Rz), the model achieved the highest evaluation indicators after 10,000 epochs as shown in Table 8. Specifically, the accuracy index was 91% for predicting the cutting force value, and 80% for predicting the surface roughness of turning products. In this case, the model’s prediction error was still significant because the input data through simulation cases were not large enough. The model will learn and make better predictions when the number of simulation cases is increased, which will be done in further studies.

## 4. Conclusions

In this study, the effects of several factors, including toolholder length, cutting speed, feed rate and wavelength on surface quality, were investigated through the finite element method of turning. The results showed that the toolholder length had the most significant impact on cutting force variation and surface roughness. The critical value for toolholder length in this study was determined to be 60 mm, with a corresponding surface roughness of approximately 20 µm. When the toolholder length exceeded this value, the surface roughness increased rapidly.

The study did not show a clear difference in cutting force variation or surface undulation of the finished part when considering the parameters of feed rate and cutting speed, as well as the surface wave of the workpiece. This was due to the effects of kinematics, such as inertia and gridding of the model.

The study’s results also demonstrated the potential use of machine learning tools as a convenient and accurate method for estimating surface roughness and cutting force parameters during turning. By combining simulation data with experimental data, the technology system could be characterized, and the machine learning model could be made more accurate. The author suggested that this study could be expanded to other factors and processes, such as drilling, milling, and grinding, using the neural network model as the basis for technology engineers to select appropriate machining parameters.

## Figures and Tables

**Table 1:** The workpiece, tool and cutting parameters.

	Parameters	Values
Workpiece	Material	Al_6061
	Length	5 mm
	Wavelength	0–0.2 mm
Tool	Material	Carbide
	Rake angle	5°
	Relief angle	10°
	Cutting edge radius	0.2 mm
	Toolholder length	10–100 mm
Cutting	Speed	40–125 m/min
	Feed rate	0.08–0.3 mm/rev
	Depth of dut (DOC)	1 mm

**Table 2:** The relationship among cutting force (A), surface roughness (Rz) and toolholder length (l).

No.	l (mm)	A (N)	Rz (µm)
1	10	10	0.4
2	15	10	0.3
3	20	12	1.2
4	25	10	1.3
5	30	5	0.3
6	35	10	2.7
7	40	15	4.8
8	45	22	4.7
9	50	40	5.9
10	55	70	10.5
11	60	95	12.8
12	65	110	16.4
13	70	120	20.5
14	75	130	30.6
15	80	180	36
16	85	150	43.5
17	90	170	52.3
18	95	180	70.8
19	100	170	70.9

**Table 3:** The relationship among cutting force (A), surface roughness (Rz) and wavelength (s).

No.	s (mm)	A (N)	Rz (µm)
1	0	170	33.8
2	0.01	165	34.2
3	0.02	160	34.5
4	0.03	160	32
5	0.04	155	37.2
6	0.05	162	35.4
7	0.06	170	38
8	0.07	170	38.9
9	0.08	150	34.9
10	0.09	140	29.9
11	0.1	180	38.2
12	0.11	128	25.2
13	0.12	145	33.9
14	0.13	145	37.3
15	0.14	158	32.4
16	0.15	130	28.2
17	0.16	135	31.3
18	0.17	140	29.3
19	0.18	140	28.6
20	0.19	135	33.3
21	0.2	165	41

**Table 4:** The relationship among cutting force (A), surface roughness (Rz) and cutting speed (v).

No.	v (m/min)	A (N)	Rz (µm)
1	40	105	12.8
2	45	111	14.8
3	50	105	12.3
4	55	120	15.5
5	60	115	16.8
6	65	138	10.4
7	70	120	16.2
8	75	104	15
9	80	125	13.2
10	85	130	15.9
11	90	150	10.6
12	95	125	15.9
13	100	130	15
14	105	128	16
15	110	90	11.8
16	115	115	14.7
17	120	110	13.7
18	125	90	10.3

**Table 5:** The relationship among cutting force (A), surface roughness (Rz) and feed rate (f).

No.	f (mm)	A (N)	Rz (µm)
1	0.08	120	31.1
2	0.09	130	29.3
3	0.1	140	31.6
4	0.11	140	31.7
5	0.12	150	33.7
6	0.13	150	37.7
7	0.14	140	36.5
8	0.15	170	36.7
9	0.16	150	36.7
10	0.17	150	32.8
11	0.18	175	36.8
12	0.19	150	39.2
13	0.2	160	33
14	0.21	170	42.7
15	0.22	170	34.7
16	0.23	170	32.9
17	0.24	180	41.3
18	0.25	165	35.5
19	0.26	175	35.7
20	0.27	185	39.7
21	0.28	160	41.4
22	0.29	165	39.5
23	0.3	190	41.3

**Table 6:** The relationship among feed rate (f), wavelength (s), cutting speed (v), toolholder length (l), cutting force (A) and surface roughness (Rz).

No.	f (mm)	s (mm)	v (m/min)	l (mm)	A (N)	Rz (µm)
1	0.09	0.00	40	40	12	0.2
2	0.09	0.05	60	50	35	8.4
3	0.09	0.10	80	60	95	12.9
4	0.09	0.15	100	70	115	20.4
5	0.09	0.20	120	80	120	27.7
6	0.12	0.00	60	60	170	21.6
7	0.12	0.05	80	70	137	25.6
8	0.12	0.10	100	80	130	28.8
9	0.12	0.15	120	40	10	38.6
10	0.12	0.20	40	50	20	7.4
11	0.15	0.00	80	80	150	32.3
12	0.15	0.05	100	40	20	1.7
13	0.15	0.10	120	50	30	5.9
14	0.15	0.15	40	60	100	16.2
15	0.15	0.20	60	70	135	22.9
16	0.18	0.00	100	50	15	5.4
17	0.18	0.05	120	60	60	10.9
18	0.18	0.10	40	70	150	11.0
19	0.18	0.15	60	80	200	32.8
20	0.18	0.20	80	40	7	3.7
21	0.21	0.00	120	70	160	28.4
22	0.21	0.05	40	80	180	44.3
23	0.21	0.10	60	40	18	7.2
24	0.21	0.15	80	50	30	10.4
25	0.21	0.20	100	60	100	14.2

**Table 7:** Response table for S/N ratio.

Level	f	s	v	l
1	0.013973	0.017627	0.015890	0.003380
2	0.017512	0.018250	0.018635	0.007566
3	0.015858	0.013222	0.017054	0.015220
4	0.012817	0.016781	0.014185	0.021735
5	0.020970	0.015250	0.015366	0.033229
Delta	0.008153	0.005028	0.004450	0.029849
Rank	2	3	4	1

**Table 8:** The relationship between technological parameters and surface roughness based on the machine learning model.

	l	s	v	f	A	Rz
0	100	0.03	38.5	0.15	160	32
1	70	0.2	38.5	0.15	120	20.5
2	100	0.04	38.5	0.15	155	37.2
3	100	0.1	38.5	0.15	180	38.2
4	80	0.2	38.5	0.12	150	33.7
...	...	...	...	...	...	...
101	85	0.2	38.5	0.15	150	43.5
102	80	0.2	38.5	0.08	120	31.1
103	80	0.2	38.5	0.28	160	41.4
104	80	0.2	38.5	0.24	180	41.3
105	80	0.2	38.5	0.1	140	31.6

## Data Availability

The data used to support the findings of this study are available from the corresponding author upon request.
